# Supplementary material for: Are quality assessments in science affected by anchoring effects? The proposal of a follow-up study
Source: PLoS One. 2023 Nov 1;18(11):e0293663. doi: 10.1371/journal.pone.0293663 (PMC10619841; doi:10.1371/journal.pone.0293663)
Supplement: S1 Appendix — (DOCX) [file pone.0293663.s001.docx]

# Appendix

## Cover letter

**Invitation: Survey on quality assessments of scientific articles**

Department of Sociology
Ludwig-Maximilians-Universität Munich

and

Science Policy and Strategy Department
Administrative Headquarters of the Max Planck Society, Munich


Dear Sir or Madam,

the Department of Sociology (Ludwig-Maximilians-Universität Munich) and the Science Policy and Strategy Department (Max Planck Society) would like to invite you to take part in a quick (5-10 minute) survey about how researchers reference existing work when writing papers. Although citations and related metrics like the h-index are widely used in academia to evaluate research and allocate resources, citation decisions on which the metrics are based are poorly understood.

In a common research project on citing decisions, we have selected one of your papers and want to ask you about a specific publication that you cited in this paper. We selected one of your papers because you published an article in recent years in which you cited a paper that we included in our study. Your answers to the cited publication will help us (and the broader scientific community) to assess the validity and limitations of existing ways of evaluating and ranking scientific works, and possibly to develop superior alternatives.

***DEPENDING ON EXPERIMENTAL GROUP

One goal of our study is to examine whether certain numbers presented to respondents affect their quality assessments of papers. Research within the anchoring-and-adjustment heuristic framework revealed that initial values are used by humans as starting points in estimations of various objects. In this study, therefore, we present you THE ACCESS CODE BELOW / INFORMATION ON THE CITATION COUNTS THE CITED REFERENCE RECEIVED WHICH IS PRESENTED IN THE QUESTIONNAIRE. ***

Please follow this link to the survey or copy and paste the URL below into your internet browser:

URL

***DEPENDING ON EXPERIMENTAL GROUP:

Please enter this code at the beginning of the questionnaire: CODE

***

Your answers will only be analyzed in anonymized form. We will delete your email address and your name once the survey is completed (this information will not be linked to your answers). We collected email addresses and names of corresponding authors from publicly available sources. The information can be found in the Web of Science database (see https://clarivate.com/products/webofscience) provided by Clarivate (see https://clarivate.com). No other personal data was or will be collected.

Many thanks,

Lutz Bornmann (bornmann@gv.mpg.de, www.researchgate.net/profile/Lutz-Bornmann)
Christian Ganser (christian.ganser@soziologie.uni-muenchen.de, www.en.ls4.soziologie.lmu.de/ganser)
